# Inhibitors of mTOR in aging and cancer

**DOI:** 10.18632/oncotarget.6878

**Published:** 2015-12-31

**Authors:** Alexander Zhavoronkov

**Affiliations:** The Biogerontology Research Foundation, Chenoweth House, Trevissome Park, Truro, UK

**Keywords:** mTOR, Rapamycin, mTOR inhibitors, gerosuppression

mTOR (target of Rapamycin) was initially found as a target of rapamycin. Rapamycin was developed as anti-cancer drug with mild effect and lacking side-effects. It causes cytostatic inhibition of proliferation and inhibits HIF-1. Only superpharmacological ( very high doses) concentration of Rapamycin and other rapalogs (for example, everolimus) causes cytotoxic effect that kills cells. In part it was attributed to the inhibition of 4EBP [1–2], rapamycin-insensitive target. Also, rapalogs, including Rapamycin, do not inhibit mTOR complex 2 (mTORC2). This target is considered to be important for cytotoxic, killing effect of dual (pan) mTOR inhibitors, named as Mtorins to cover the group of all pan-Mtorins, including Torin1, Torin 2, AZD8055, PP242, KU-006379 and GSK1059615 and all other Mtorins.

Some of Mtorins are currently undergoing phase-2 clinical trials. Except special malignancies their effect is not spectacular, albeit better effects than rapalogs might be expected. Rapalogs themselves and in combination with other drugs are effective against some group of tumors. Nobody considers rapalogs as a miracle in cancer treatment. This was predicted in several publications, including by Blagosklonny M.V. in (2008-2014) [3–5]. The inhibitors are not anticancer drugs, they are antiaging ones. They prevent future cancer by slow down aging. Rapalogs effectively inhibit aging in human cells, yeast, Drosophila (fly), worms, mice, monkeys, and as it will be shown soon, in humans [5–7]. Not surprisingly they prevent and delay cancer in mice (sometimes as much as for 90%). There is a mechanistic link between the suppression of senescence (gerosuppression) and suppression of cancer (cancer suppression). Rapalogs are both gero- and cancer suppressants. Their gerosuppression results in cancer suppression. Some other agents and conditions that suppress cancer, like contact inhibition, also inhibit mTOR, acting as rapamycin [8]. It is so universal that it was suggested that dual inbitors of mTOR (Mtorins) will also be gerosuppressors (Torin1, Torin 2, AZD8055, PP242, KU-006379 and GSK1059615). It was shown that at low doses they act as Rapamycin or other rapalogs [9]. They preferentially inhibit Rapamycin-sensitive function of mTORC1 such as inhibition of S6 kinase and S6. At higher doses they inhibit phosphorylation of 4E-BP, which is Rapamycin insensitive in the most conditions [6]. At higher doses they even inhibit mTORC2 complex as measured by inhibition of phosphorylation of Akt. Therefore, at the range of concentrations Mtorins reproduce the effect of Rapamycin (rapalogs), but also slightly inhibit rapalogs –insensitive functions of Rapamycin. This gives an additional advantage compared with rapalogs. And these drugs have only minimal cytostatic and not cytotoxic effect. They do not kill cells. Minimal side-effects can be expected at these doses. These doses will be called optimal gero-suppressive. At optimal gero-suppressive doses Mtorins effectively decrease cell size, normalize cell morphology and decrease beta-Gal cell staining when added together with senescence inducing agents [9]. When senescence inducing agents and Mtorins are washed out, the cells can restart re-proliferation. They eventually will become normal cells. While in the absence of Mtorins, the cells will become flat, beta-gal positive.

So Mtorins demonstrate themselves as gerosuppressants. Future animal studies are needed to select optimal *in vivo* doses compared with optimal *in vitro* concentration. We expect that Mtorins will show true anti-cancer effect, not by directly killing anti-cancer cells, but by slowing down aging.

It was expected that at anti-cancer doses Mtorins will have side-effects and some side-effect were detected in clinical trials, although these side-effects were tolerable. At 20 times lower concentration we expect are low or no side-effects. Clinical trials are important and should be provided simultaneously in mice and humans, given that some Mtorins have reached phase-2 clinical trials.
